# Public health system integration of avoidable blindness screening and management, India

**DOI:** 10.2471/BLT.18.212167

**Published:** 2018-08-27

**Authors:** Venkata SM Gudlavalleti, Rajan Shukla, Tripura Batchu, Bala Vidyadhar S Malladi, Clare Gilbert

**Affiliations:** a Indian Institute of Public Health, Public Health Foundation of India, Kavuri Hills, Madhapur, Hyderabad 500 033,Telangana, India; b Clinical Research Department, London School of Hygiene and Tropical Medicine, London, England.

## Abstract

In India, 73 million people have diabetes and 3.5 million infants are born preterm. Without timely screening, there is a risk of visual loss due to diabetic retinopathy and retinopathy of prematurity in these two groups, respectively. Both conditions are emerging causes of visual impairment in India but there is no public health programme for screening or management. Pilot projects were initiated in 2014 to integrate the screening and management of these conditions into existing public health systems, particularly in rural communities and their referral networks. The World Health Organization’s health systems framework was used to develop the projects and strategies were developed with all stakeholders, including the government. Both projects involved hub-and-spoke models of care units around medical schools. For diabetic retinopathy, screening was established at primary health-care facilities and treatment was provided at district hospitals. For retinopathy of prematurity, screening was integrated into sick newborn care units at the district level and treatment facilities were improved at the closest publically funded medical schools. In the first two years, there were substantial improvements in awareness, screening, treatment and partnership between stakeholders, and changes in public health policy. By March 2018, diabetic retinopathy screening was established at 50 facilities in 10 states and treatment had been improved at 10 hospitals, whereas retinopathy of prematurity screening was established at 16 sick newborn care units in district hospital in four states and treatment had been improved at six medical schools. Advocacy within state governments was critical to the success of the initiative.

## Résumé

En Inde, 73 millions de personnes sont atteintes de diabète et 3,5 millions de nourrissons naissent avant terme. Lorsque le dépistage n'est pas effectué à temps, il existe un risque de perte de la vue due à la rétinopathie diabétique dans le cas du premier groupe et à la rétinopathie du prématuré dans le cas du second. Bien que ces deux maladies soient de nouvelles causes de déficience visuelle en Inde, aucun programme de santé publique ne vise leur dépistage ou leur prise en charge. Des projets pilotes ont été lancés en 2014 pour intégrer le dépistage et la prise en charge de ces maladies dans les systèmes de santé publique existants, en particulier dans les communautés rurales et leurs réseaux d'orientation. Le cadre des systèmes de santé de l'Organisation mondiale de la Santé a été utilisé pour développer ces projets et des stratégies ont été élaborées avec toutes les parties prenantes, et notamment le gouvernement. Les deux projets impliquaient des réseaux en étoile d'unités de soins autour des écoles de médecine. Dans le cas de la rétinopathie diabétique, le dépistage a été assuré dans des établissements de soins de santé primaires, tandis que le traitement a été appliqué dans des hôpitaux de district. Dans le cas de la rétinopathie du prématuré, le dépistage a été intégré dans des unités de soins pour les pathologies néo-natales au niveau des districts et les installations de traitement ont été améliorées dans les écoles de médecine financées par l'État les plus proches. Au cours des deux premières années, des améliorations considérables ont été constatées en matière de sensibilisation, de dépistage, de traitement et de partenariat entre les parties prenantes, et des changements ont été apportés à la politique de santé publique. En mars 2018, le dépistage de la rétinopathie diabétique était assuré dans 50 établissements répartis dans 10 États et le traitement avait été amélioré dans 10 hôpitaux, tandis que le dépistage de la rétinopathie du prématuré était assuré dans 16 unités de soins pour les pathologies néo-natales dans les hôpitaux de district de quatre États et le traitement avait été amélioré dans six écoles de médecine. Un soutien au sein des administrations des États fédérés a été essentiel au succès de l'initiative.

## Resumen

En la India, 73 millones de personas son diabéticas y 3,5 millones de niños nacen prematuros. Sin exámenes de detección oportunos, existe un riesgo de pérdida de la visión por la retinopatía diabética y la retinopatía por prematuridad en estos dos grupos, respectivamente. Ambas afecciones son causas emergentes de discapacidad visual en la India, pero no existe un programa de salud pública para la detección o el tratamiento. En 2014 se iniciaron proyectos piloto para integrar el análisis y la gestión de estas condiciones en los sistemas de salud pública existentes, en particular en las comunidades rurales y sus redes de derivación. Se utilizó el marco de sistemas de salud de la Organización Mundial de la Salud para desarrollar los proyectos y se desarrollaron estrategias con todas las partes interesadas, incluido el gobierno. En ambos proyectos se utilizaron modelos de unidades de atención centralizados en torno a las facultades de medicina. En cuanto a la retinopatía diabética, se establecieron exámenes de detección en los centros de atención primaria y se ofreció tratamiento en los hospitales de distrito. En cuanto a la retinopatía por prematuridad, los exámenes de detección se integraron en las unidades de atención neonatal a nivel de distrito y se mejoraron las instalaciones de tratamiento en las facultades de medicina más cercanas financiadas con fondos públicos. En los dos primeros años se produjeron mejoras sustanciales en la sensibilización, la detección, el tratamiento y la asociación entre las partes interesadas, así como cambios en la política de salud pública. Para marzo de 2018, se establecieron exámenes de detección de retinopatía diabética en 50 establecimientos de 10 estados y se mejoró el tratamiento en 10 hospitales, mientras que se establecieron exámenes de detección de retinopatía por prematuridad en 16 unidades de atención neonatal en hospitales de distrito de cuatro estados y se mejoró el tratamiento en seis facultades de medicina. La promoción dentro de los gobiernos estatales fue fundamental para el éxito de la iniciativa.

## ملخص

يعاني 73 مليون شخصاً بالهند من مرض السكري، كما أن هناك 3.5 مليون رضيعاً مبتسراً (وُلد قبل الأوان). في حالة عدم الفحص في الوقت المناسب، يكون هناك خطر لفقدان البصر بسبب اعتلال الشبكية السكري، واعتلال الشبكية لدى المبتسرين، في هاتين المجموعتين على التوالي. كلا الحالتين هي أسباب ناشئة لضعف البصر في الهند، إلا أنه لا يوجد برنامج للصحة العامة يتولى الفحص أو الإدارة. تم تدشين مشروعات رائدة في عام 2014 لدمج عمليتي فحص وإدارة هذه الحالات في نظم الصحة العامة القائمة، وخاصة في المجتمعات الريفية وشبكات الإحالة الخاصة بها. تم استخدم إطار النظم الصحية التابع لمنظمة الصحة العالمية، لتطوير المشاريع، كما تم وضع استراتيجيات مع كل الجهات المعنية، بما في ذلك الحكومة. اشتمل كلا المشروعين على نماذج محورية لوحدات الرعاية في أنحاء كليات الطب. بالنسبة للاعتلال الشبكي السكري، تم إجراء الفحص في مرافق الرعاية الصحية الأولية، كما تم تقديم العلاج في مستشفيات المقاطعات. أما بالنسبة للاعتلال الشبكي لدى المواليد المبتسرين، فقد تم دمج الفحص في وحدات رعاية حديثي الولادة المرضى على مستوى المقاطعة، كما تم تحسين مرافق العلاج في أقرب كليات الطب الممولة من القطاع العام. في العامين الأولين، كانت هناك تحسينات كبيرة في الوعي والفحص والعلاج والشراكة بين الجهات المعنية، بالإضافة إلى تغييرات في سياسة الصحة العامة. وبحلول مارس/آذار 2018، أصبح الكشف عن اعتلال الشبكية السكري متوافراً في 50 مرفقاً في 10 ولايات، كما تم تحسين العلاج في 10 مستشفيات، بينما أصبح فحص اعتلال الشبكية لدى المبتسرين متوفراً في 16 وحدة لرعاية حديثي الولادة المرضى في مستشفى المقاطعة في أربع ولايات، وتم تحسين العلاج في ست من كليات الطب. كانت جهود الدعم داخل حكومات الولايات ضرورية لنجاح المبادرة.

## 摘要

在印度，七千三百万人患有糖尿病，三百五十万婴儿是早产儿。如果未及时筛查，以上两组人群分别会有糖尿病性视网膜病变和早产儿视网膜病变，从而导致视力丧失。这两种情况都是印度人民视力受损的新兴原因，然而印度本国并没有用于筛查或管理的公共卫生计划。试点项目于 2014 年启动，并将这些情况的筛选和管理纳入现有公共卫生系统，特别是在农村社区及其转诊网络中。应用世界卫生组织的卫生系统框架来制定项目，并与包括政府在内的所有利益攸关方共同制定战略。这两个项目都包括了以医学院为中心、医疗单位为周边的轴辐式模型。针对糖尿病性视网膜病变，在初级卫生保健机构进行筛查，并在地区医院给予治疗。针对早产儿视网膜病变，筛查被纳入地区级别的患病新生儿护理单位，并对位置最近的公立医学院的治疗设施进行了改善。在最初两年内，意识、筛查、治疗和利益攸关方间的合作关系以及公共卫生政策变化都有了实质性的改善。截至 2018 年 3 月，10 个邦的 50 家机构中建立了糖尿病视网膜病变筛查，10 家医院的治疗得以改善，并在 4 个邦地区医院的 16 个患病新生儿护理单位建立了早产儿视网膜病变筛查，6 家医学院的治疗得以改善。邦政府内部宣传对该倡议的成功至关重要。

## Резюме

В Индии 73 миллиона человек страдают диабетом и 3,5 миллиона младенцев рождаются преждевременно. Без своевременного скрининга в этих двух группах существует риск потери зрения вследствие диабетической ретинопатии и ретинопатии недоношенных. Оба эти состояния являются растущими причинами нарушения зрения в Индии, но в этой стране нет никакой соответствующей программы общественного здравоохранения для скрининга или лечения. В 2014 году были начаты экспериментальные проекты по интеграции скрининга и лечения этих состояний в существующие системы общественного здравоохранения, особенно в сельских общинах и сети медицинских учреждений, находящихся в сельской местности. Для разработки проектов за основу была взята структура системы здравоохранения, рекомендованная Всемирной организацией здравоохранения, и стратегии были разработаны совместно со всеми заинтересованными сторонами, включая правительство. Оба проекта включали в себя модель системы здравоохранения, представляющую собой разветвленную сеть медицинских учреждений, расположенных на базе медицинских учебных заведений. Скрининг по поводу диабетической ретинопатии был внедрен в учреждениях первичной медико-санитарной помощи, а лечение проводилось в районных больницах. Скрининг по поводу ретинопатии недоношенных был интегрирован в неонатологические отделения больниц на районном уровне, а также были усовершенствованы лечебные учреждения в ближайших государственных медицинских учебных заведениях. В течение первых двух лет произошли существенные улучшения в информировании, скрининге, лечении и партнерстве между заинтересованными сторонами, а также изменения в политике общественного здравоохранения. К марту 2018 года скрининг по поводу диабетической ретинопатии был внедрен на 50 объектах в 10 штатах, лечение было усовершенствовано в 10 больницах, тогда как скрининг в связи с ретинопатией недоношенных был внедрен в 16 неонатологических отделениях районных больниц в четырех штатах, а лечение было усовершенствовано в шести медицинских учебных заведениях. Поддержка правительств штатов имела решающее значение для успеха инициативы.

## Introduction

The Vision Loss Expert Group of the Global Burden of Disease study reported that globally there were 36 million blind people in 2015, of whom 11.7 million (32.5%) lived in South Asia.^1^ In addition, they found that 81.2% of all blindness was avoidable,^2^ though less than 50% may be avoidable in children.^3^ Internationally, blindness due to avoidable retinal disease is increasing.^4^ In particular, India is experiencing a so-called third epidemic of blindness caused by retinopathy of prematurity,^5^ in addition to a dramatic increase in diabetic retinopathy.^6^^–^^8^ The causes are, respectively, increased survival of preterm infants due to expanded provision of neonatal care and increased diabetes linked to an ageing population and lifestyle changes.^9^^–^^12^

In 2010, an estimated 32 200 infants globally had visual loss from retinopathy of prematurity.^13^ In India, the figure that year was 5000 (i.e. 15% of the global estimate) among the 3.52 million preterm births in the country (i.e. 23.6% of 15 million preterm births globally).^9^^,^^14^^,^^15^ Between 10 and 47% of premature babies born under 32 weeks’ gestation who survive neonatal care develop retinopathy of prematurity^16^^,^^17^ and up to 15% of survivors require treatment for sight-threatening retinopathy.^5^^,^^9^ Risk factors for the condition are earlier prematurity, inadequate monitoring of supplemental oxygen, respiratory distress syndrome, anaemia and sepsis.^13^^,^^18^ The risk of blindness can be reduced by minimizing preterm births, better neonatal care from birth and timely screening of infants at risk, with urgent treatment for those who develop sight-threatening retinopathy of prematurity.^19^ Screening and treatment of the condition have been reported to be highly cost–effective.^20^^,^^21^

Globally, 35.4% of people with diabetes mellitus were estimated to have diabetic retinopathy in 2012 and 11.7% had sight-threatening disease.^22^ In 2017, 73 of the 451 million people with diabetes worldwide (16.2%) lived in India.^23^ Studies carried out in the country over the last decade reported that diabetic retinopathy prevalence were approximately 9.6% in rural areas^24^ and 18.0% (255/1414) in urban areas;^7^ the rates of sight-threatening retinopathy in the two areas were 3.8% (45/1190) and 6.6% (39/592), respectively.^8^^,^^25^ Risk factors for diabetic retinopathy include longer duration of diabetes, poor diabetes control and hypertension. Evidence from high-income countries indicates that the risk of visual loss from diabetic retinopathy can be reduced by strategies such as better control of blood glucose and hypertension and regular screening to detect individuals with sight-threatening diabetic retinopathy, followed by confirmatory diagnosis and appropriate management.^26^

## Current situation in India

India has a federal structure of governance with an elected national government and 28 state governments. Although the national government oversees the formulation of policies, medical education and health programmes, public health provision is the responsibility of state governments, who decide how to implement health initiatives. Districts (average population: 1.9 million) are the smallest administrative units of states and health programmes are implemented locally by district health societies.

Services for sick and preterm infants in India have been rapidly expanded over the last decade.^27^ Since 2008, the national government has established 525 district-level special newborn care units, which cover 414 districts. The remaining 208 districts are expected to be covered soon.^28^ However, the quality of neonatal care varies and current policies do not include the control of retinopathy of prematurity.^29^ Treatment of the condition has mostly been undertaken by the not-for-profit eye sector – provision by the government sector is minimal. Although there are no formal government guidelines for retinopathy of prematurity screening, the national government has mandated that preterm infants with a birth weight under 2000 g be screened for retinopathy of prematurity no later than 30 days after birth.^5^ Babies admitted to neonatal units should be screened, with systems to ensure high coverage.".

Retinopathy of prematurity is included in three national, vertical programmes: (i) the National Child Health Programme recommends intensive neonatal care for all preterm and low-birth-weight infants, as indicated in the 2014 Newborn Action Plan;^30^ (ii) Rashtriya Bal Swasthya Karyakram provides screening and early intervention services for children with specific conditions, including retinopathy of prematurity; and (iii) the National Programme for Control of Blindness is involved in preventing avoidable blindness, including increasing the ability of medical schools to screen and treat retinopathy of prematurity.

In India, there is no systematic screening for the complications of diabetes, such as diabetic retinopathy, despite a rapid increase in the incidence of the disease. Retinal examinations are performed opportunistically when people with diabetes visit an eye facility, though often only after vision loss. Diabetic retinopathy is included in two national, vertical programmes: (i) the National Programme for Prevention and Control of Cardiovascular Diseases, Cancers, Diabetes and Stroke, established in 2010, is responsible for all noncommunicable diseases – programme personnel are responsible for the identification and registration of people with diabetes and for providing monthly medication; and (ii) the National Programme for Control of Blindness assists in the diagnosis and management of diabetic retinopathy.

For both diabetic retinopathy and retinopathy of prematurity, there is a lack of synergy between these vertical programmes, little collaboration between eye-care professionals and professionals who manage preterm infants or diabetes, and limited awareness among families of the implications of prematurity and diabetes for vision loss. In addition, the majority of people affected live in rural areas, are relatively poor and uneducated and cannot afford private health care.^31^ The provision of universal eye health, with an emphasis on better access for the poorest individuals, is crucial.^32^ In India, this will involve integrating strategies for the control of diabetic retinopathy and retinopathy of prematurity into the existing health-care system.

Health systems and policies play critical roles in determining how health services are delivered and used and, consequently, influence health outcomes.^33^ In the early 2010s in India, there was a need for specific policies and for advocacy to prevent vision loss from retinopathy of prematurity and diabetic retinopathy, both important causes of avoidable blindness. Although the two conditions present different challenges, because of differences in their time of onset and severity, in the age groups affected and in the urgency and level of care required, they can be prevented, detected and managed using similar strategies, which could be embedded in policies. The recent, government-supported, expansion of the public health system, particularly in rural areas, provided an opportunity to explore ways of meeting these challenges.^33^

## Pilot projects

**Fig. 1:**
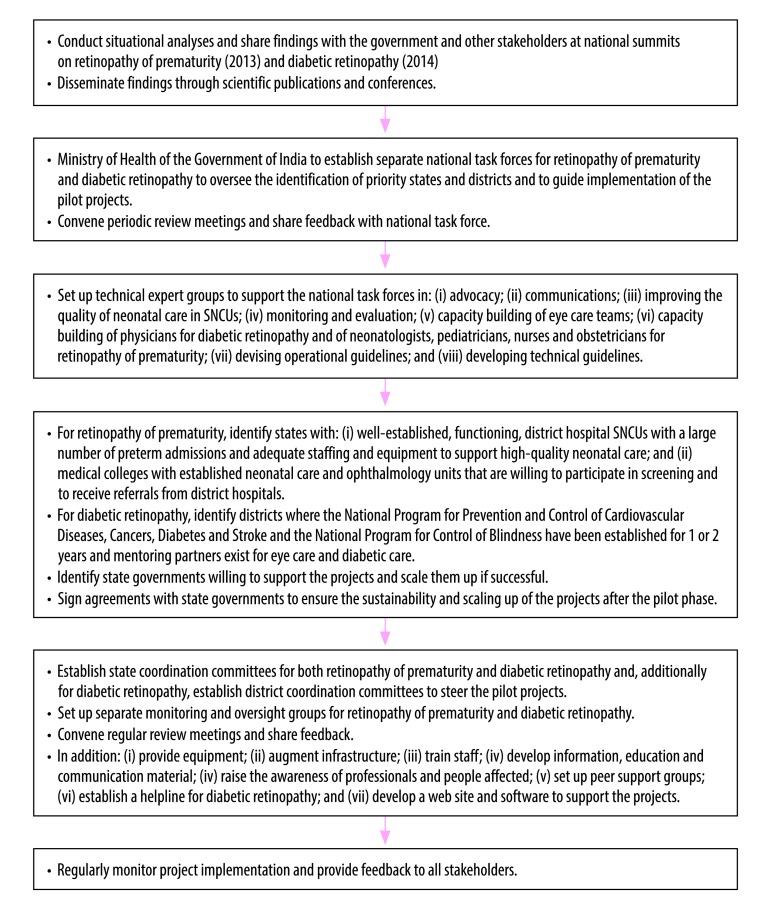
Flow diagram, retinopathy of prematurity and diabetic retinopathy pilot projects, India, 2014–2018

Since 2014, the public health system in India has been engaged in pilot projects on the control of retinopathy of prematurity and diabetic retinopathy (Table 1). The projects were both supported by the Queen Elizabeth Diamond Jubilee Trust and were managed by the Public Health Foundation of India. The overall goal was to develop models of care to reduce avoidable blindness from the two conditions by integrating screening and treatment into the public health system at every level in a way that was scalable and sustainable. There was a need for transformational change and similar approaches were adopted for the two conditions (Fig. 1).

**Table 1:** Diabetic retinopathy and retinopathy of prematurity pilot project initiatives at public health-care facilities, India, 2014–2018

Health-care level	Health-care facility and personnel	Pilot project initiative
Primary – first point of contact	Subcentres: 1 per 5 000 population, each with 2 health workers and an additional female health worker (155 069 subcentres were operating in 2017); in each village, there was 1 accredited social health activist who acted as the community link for health programmes for every 1 000 people	Diabetic retinopathy: (i) annual screening for diabetes implemented at the community level, with people with a high random blood sugar level referred to medically qualified personnel at primary health centres. Diabetic retinopathy and retinopathy of prematurity: (i) accredited social health activists were made aware of the need to screen people with diabetes for diabetic retinopathy; and (ii) accredited social health activists and female health workers were made aware of retinopathy of prematurity
Primary – first point of contact with medically qualified personnel	Primary health centres (1 per 30 000 population) that provide integrated health-care services for health promotion, prevention and cure and each have 1–2 medical officers supported by 15 paramedical staff (25 354 were operating in 2017)	Diabetic retinopathy: (i) register of people with diabetes established; (ii) diabetes drugs provided for free; and (iii) medical staff received training on diabetic retinopathy
Secondary – first point of contact with specialists	Community health centres (1 per 100 000 population) that provide in-patient facilities, employ 4 medical officers supported by 21 paramedical personnel each, and have internal medicine, paediatric and obstetric specialists and ophthalmic assistants for refraction and vision testing and postoperative care (5 510 were operating in 2017)	Diabetic retinopathy: (i) these centres were the first formal structures embedded in the diabetic retinopathy pilot projects that had the necessary infrastructure for screening for diabetic retinopathy; (ii) tablet computers were provided to register people with diabetes who attended these centres and were receiving treatment; (iii) 1 or 2 medical officers at each centre received training on diabetic retinopathy; (iv) fundus cameras provided for diabetic retinopathy screening; and (v) ophthalmic assistants underwent training in image capture and storage, with an emphasis on the initial grading of images
Secondary – referral pathway	District health centres (1 per 1.0–1.5 million population) that provide advanced care in most medical specialties and have an ophthalmologist, ophthalmic assistant, paediatrician and obstetrician (600 were operating in 2017)	Retinopathy of prematurity: (i) these centres provided the points of integration for retinopathy of prematurity screening; (ii) quality improvements were implemented in neonatal teams; (iii) staff and ophthalmologists in neonatal care units underwent training in setting up, managing and undertaking screening for retinopathy of prematurity; (iv) 1–2 ophthalmologists at each centre underwent training in both retinopathy of prematurity screening and laser treatment; and (v) equipment was provided for screening (i.e. indirect ophthalmoscopes) and laser treatment. Diabetic retinopathy: (i) ophthalmologists trained to diagnose diabetic retinopathy and treat the condition using lasers and anti-vascular endothelial growth factors; and (ii) equipment provided for treatment
Tertiary – specialized referral pathway	Medical schools that are managed by the directorate of medical education and provide specialized medical care, including rehabilitation	Retinopathy of prematurity and diabetic retinopathy: (i) medical schools acted as mentoring partners on diabetic retinopathy and retinopathy of prematurity for staff in neighbouring districts. Retinopathy of prematurity: (i) ophthalmologists were trained to screen for the condition and provided with equipment, such as lasers, for treatment

SNCU: sick newborn care unit.

With the assistance of the national government, two separate national task forces were established. They comprised professional experts, public health personnel, staff responsible for national government programmes and staff from nongovernmental organizations and other agencies, such as the United Nations Children's Fund (UNICEF). The task forces identified key strategies and the processes to be implemented and have important coordinating and oversight roles. States that expressed a willingness to participate in the pilot projects and, subsequently, to extend activities to others areas of the state were prioritized, if they met feasibility criteria. The task forces also identified performance indicators, established a monitoring matrix for reporting and created a dedicated online database for each of the two conditions. Each task force set up technical expert groups to support decision-making and project implementation. The objectives and expected outcomes of the pilot projects are summarized in Box 1.

Box 1 **Objectives and expected outcomes, retinopathy of prematurity and diabetic retinopathy pilot projects, India, 2014–2018**

**Retinopathy of prematurity pilot project**

Objectives

Advocacy for policy change at national and state government levels.

Development of teaching modules and methods for quality improvement training for neonatal care teams.

Development of national guidelines for the prevention of blindness from retinopathy of prematurity.

Increased capacity of eye care and neonatal care teams in the public health sector.

Identification, implementation and evaluation of integrated models for the screening and treatment of retinopathy of prematurity at 3 or 4 district hospitals and a medical school in each of 4 or 5 Indian states.

Feasibility assessment of the pilot project.

Identification of lessons learnt to help implement screening and treatment in non-participating locations.

Expected outcomes

By the end of 2017, government agencies should have issued at least two documents or orders on policy changes affecting retinopathy of prematurity.

By the end of 2018, at least 50% of infants with a birth weight < 2000 g treated in special newborn care units should have been screened for retinopathy of prematurity within 30 days of birth.

By the end of 2018, the proportion of infants with severe retinopathy of prematurity treated within 48 hours of diagnosis should have increased by 25%.

**Diabetic retinopathy pilot project**

Objectives

Advocacy for policy change at national and state government levels.

Development of national guidelines for diabetic retinopathy screening and management.

Increased capacity of eye care and physician teams.

Improvements in the knowledge and skills of people with diabetes and their families on managing diabetes and its risk factors, including diabetic retinopathy.

Identification, implementation and evaluation of integrated models of care for diabetes and its eye complications in 8–10 pilot districts (approximate population per district: 5 million) in a total of 10 states.

Feasibility assessment of the pilot project.

Identification of lessons learnt to help implement screening and treatment in non-participating locations.

Expected outcomes

By the end of 2018, the proportion of people with diabetes screened for diabetic retinopathy should have increased by 50%.

By the end of 2019, the proportion of people diagnosed with diabetes being screened annually should have increased by 50%.

By the end of 2018, 75% of registered people with diabetes should be aware of the risk of visual impairment from diabetic retinopathy.

### Retinopathy of prematurity

In 2013, a national summit on retinopathy of prematurity identified major gaps in services and delegates recommended four main strategies to deal with them: (i) improve the ability of neonatal care units to reduce the risk of sight-threatening retinopathy of prematurity; (ii) integrate the screening and treatment of retinopathy of prematurity into child health services; (iii) establish a hub-and-spoke model (comprising three or four special newborn care units around one medical school) in each state to provide screening and treatment; and (iv) establish public–private partnerships, where applicable, so that existing expertise in neonatal care and eye care can be used to mentor staff in special newborn care units and eye-care professionals in the public health sector.

The national task force for retinopathy of prematurity started work in 2014. A situation analysis was carried out in nine states and state programme officials underwent awareness training. The existing national database for special newborn care units was consulted to obtain data. Such as on the number of preterm infants admitted, and site visits provided additional information on capacity, infrastructure, equipment, facilities, personnel and staff capabilities at public sector special newborn care units and eye-care service providers in local district hospitals and medical schools. The task force identified five states where the retinopathy of prematurity pilot project could be implemented based on preterm birth admission rates, survival rates, infrastructure, the commitment of the state government and the willingness of neonatal and eye-care teams to participate. In addition, mentoring institutions for eye care and neonatal care were identified in each state. Finally, agreements were signed with four states and strategies approved by state coordination committees were implemented. It was agreed that screening for retinopathy of prematurity would be embedded within special newborn care units and neonatal intensive care units, with responsibility shared between neonatal care and eye-care personnel. Rashtriya Bal Swasthya Karyakram was responsible for coordinating screening, referral and long-term follow-up. A technical expert group developed a comprehensive quality improvement package, which was being rolled out at the time of writing.

The retinopathy of prematurity pilot project aimed to promote programme coordination, professional collaboration and partnership: (i) programme coordination between the National Child Health Programme, Rashtriya Bal Swasthya Karyakram and the National Programme for Control of Blindness; (ii) professional collaboration between ophthalmologists and neonatal care teams; and (iii) partnerships between public health services, nongovernmental organizations and the private sector. Substantial investment was also made in developing educational material to promote the best neonatal care practices for ensuring infant survival without retinopathy of prematurity. The World Health Organization’s (WHO’s) health system framework was used in developing the pilot project (Table 2).^34^

**Table 2:** Development of retinopathy of prematurity and diabetic retinopathy pilot projects using WHO’s health systems framework, India, 2014–2018

Aspect of health systems framework	Retinopathy of prematurity pilot project		Diabetic retinopathy pilot project	
	Activities envisaged	Outcomes achieved by March 2018	Activities envisaged	Outcomes achieved by March 2018
**Building block**				
Leadership and governance	(i) establish a national task force and state coordination committees to steer the project; (ii) establish technical expert groups for individual project components; and (iii) identify strategies for implementation	(i) national task force met 4 times; (ii) 9 state coordination committee meetings held; (iii) 22 technical expert group meetings held; and (iv) 4 meetings held between project management team and national health secretary	(i) establish a national task force and state and district coordination committees to steer the project; (ii) establish technical expert groups for individual project components; and (iii) identify strategies for implementation	(i) national task force met 5 times; (ii) 7 state coordination committee and 5 district coordination committee meetings held; (iii) 19 technical expert group meetings held; and (iv) 4 meetings held between project management team and national health secretary
Health workforce	(i) improve the capabilities of paediatricians, nurses, ophthalmologists and support personnel	(i) 21 ophthalmologists trained to screen for retinopathy of prematurity; (ii) 5 ophthalmologists trained in laser treatment; (iii) 21 paediatricians and 48 nurses trained in improving neonatal care and preventing retinopathy of prematurity; (iv) 19 nurse educators trained in improving the quality of neonatal care; and (v) staff in 16 district early intervention centres trained in improving neonatal care and reducing the risk retinopathy of prematurity	(i) improve the capabilities of physicians, ophthalmologists, ophthalmic assistants and primary health-care personnel	(i) 127 personnel trained in diabetic retinopathy screening; (ii) 27 ophthalmologists trained in laser treatment; (iii) 3 153 health support staff educated about diabetic retinopathy; (iv) 326 physicians educated about risk factors and early screening for diabetic retinopathy; and (v) e-learning modules developed for physicians
Service delivery	(i) identify 4 states each with 6 medical schools and 12 district hospital SNCUs suitable for participation; and (ii) establish infrastructure for the screening and treatment of retinopathy of prematurity	(i) 5 971 preterm infants screened; and (ii) 185 infants treated for retinopathy of prematurity	(i) identify 10 districts suitable for participation; (ii) establish infrastructure for screening and managing diabetes and its risk factors for complications at noncommunicable disease clinics; and (iii) augment facilities for managing diabetic retinopathy at the district level	(i) 34 550 people with diabetes screened for diabetic retinopathy; (ii) 1 601 people with diabetes treated for diabetic retinopathy; and (iii) 15 diabetes support groups established
Medical products and technologies	(i) provide all pilot district hospitals with an indirect ophthalmoscope for screening; (ii) provide 7 medical schools with laser treatment systems; and (iii) establish simulation laboratories for quality improvement training at mentoring institutions and state medical schools	(i) 14 indirect ophthalmoscopes, 6 Retcams, 8 laser treatment units, 22 Volk 20D diagnostic lenses, 8 Volk 30D diagnostic lenses, 1 pulse oximeter and 1 Heine Omega 500 digital video camera provided; and (ii) dedicated open access web site set up for online training: it included 126 short webinars and videos for training to improve the quality of neonatal care and focused on preterm care for doctors, nurses and support staff	(i) install fundus cameras for screening at noncommunicable disease clinics in community health centres; (ii) install laser treatment systems at district hospitals; (iii) provide optical coherence tomography equipment at tertiary care centres in the district; and (iv) establish systems for on-the-spot and remote reporting of fundus images	(i) 50 fundus cameras provided at noncommunicable disease clinics; (ii) 10 laser treatment systems installed at district hospitals; (iii) optical coherence tomography equipment installed at 6 tertiary care facilities; (iv) dedicated web site developed to support people with diabetes and their caregivers; and (v) telephone counselling helpline established for people with diabetes
Information systems and research	Develop software for retinopathy of prematurity project management that: (i) is integrated with the SNCU online database; (ii) can record details of all infants eligible for retinopathy of prematurity screening, including when they were screened and their treatment and follow-up; (iii) facilitates referral for treatment; and (iv) serves as a repository for advocacy and communication documentation developed by, and shared with, stakeholders	(i) software has been developed that can track all infants eligible for retinopathy of prematurity screening and their treatment and follow-up and that can be used for clinical monitoring; and (ii) 4 tablet computers provided	(i) develop software for project management; (ii) register details of people with diabetes on tablet computers located at primary and secondary health-care centres; and (iii) carry out operational research	(i) 40 tablet computers with dedicated patient-tracking software installed at community health centres in 10 pilot districts; and (ii) 10 operational research projects commissioned
Health-care financing	(i) provide free retinopathy of prematurity screening and treatment at public health facilities in project areas	(i) free retinopathy of prematurity screening established in 16 SNCUs; and (ii) treatment facilities provided at 6 medical schools and 1 district hospital	(i) provide free diabetic retinopathy screening and laser treatment at public health facilities in project areas; and (ii) provide free medication for managing diabetes and associated risk factors, such as hypertension	(i) free diabetic retinopathy screening established at 50 health facilities in 10 districts; and (ii) diabetic retinopathy treatment services augmented in 10 district hospitals, at no cost to patients
**Performance**				
Access	(i) improve access to retinopathy of prematurity services in public health facilities in tier-2 cities (i.e. cities with a population of around 1 million and important regional hubs) and rural districts, which cover poor and rural populations	(i) need for poor parents to travel far for screening and to seek treatment in large cities reduced; and (ii) access to timely care provided	(i) register people with diabetes and carry out preliminary diabetic retinopathy screening at health centres in rural areas where such facilities were not previously available (these centres are predominantly used by women and poor people)	(i) 57.3% of people with diabetes screened were women; (ii) the need for people with diabetes to travel far for screening and treatment was reduced
Coverage	(i) screen all preterm infants admitted to SNCUs who are eligible for retinopathy of prematurity screening at the secondary care level in the public health system; and (ii) treat all infants with sight-threatening retinopathy of prematurity	ND	(i) include all registered people with diabetes seen at primary and secondary health-care centres in the public health system in project areas	ND
Quality	(i) monitor outcomes regularly; (ii) upgrade staff skills through personal and online training; and (iii) provide supportive supervision	ND	(i) monitor outcomes regularly; and (ii) upgrade staff skills through training	ND
Safety	(i) ensure the care provided is to an accepted standard	(i) operational safety guidelines were finalized and disseminated following consultations	(i) ensure the care provided is to an accepted standard	(i) national technical guidelines are being drafted

ND: not determined; SNCU: sick newborn care unit; WHO: World Health Organization.

### Diabetic retinopathy

In 2013, situation analyses of the management of diabetes and diabetic retinopathy was performed in the 11 most populous cities across nine states in India: it considered infrastructure, facilities, staff skills and capacity, and the perceptions of both people with diabetes and service providers.^35^ A critical finding was that, despite being aware that diabetes can affect the eyes, 45.7% of people with diabetes who attended eye clinics already had vision loss.^36^ The results of the analysis were presented at a national summit in 2014. In response to the summit declaration, the national government constituted a national task force to prepare and implement a four-year pilot project for 2015 to 2019 to reduce vision loss from diabetic retinopathy. This task force agreed that the control of blood glucose, lipid levels and of hypertension should be addressed, and a technical expert group developed an educational package for physicians on managing diabetes and diabetic retinopathy. Members of the task force stressed that shared responsibility between physicians and ophthalmologists should be fostered and that the screening of people with diabetes should be embedded within the National Programme for Prevention and Control of Cardiovascular Diseases, Cancers, Diabetes and Stroke and should take place in clinics where they are regularly managed. This was a paradigm shift.

As with the retinopathy of prematurity pilot project, the diabetic retinopathy pilot project aimed to promote programme coordination, professional collaboration and partnership in the health system: (i) programme coordination between the National Programme for Control of Blindness and the National Programme for Prevention and Control of Cardiovascular Diseases, Cancers, Diabetes and Stroke; (ii) professional collaboration between ophthalmologists and diabetic physicians; and (iii) partnerships between public health services, nongovernmental organizations and the private sector. The pilot project was based on the assumption that the integration of diabetic retinopathy screening into noncommunicable disease clinics in districts where the National Programme for Prevention and Control of Cardiovascular Diseases, Cancers, Diabetes and Stroke was operating, would be feasible and sustainable.

Strategies for implementing the pilot project were agreed in consultation with state governments at all levels. The establishment of services in the public health system was supported by mentors, who were mostly nongovernmental organizations. Ten districts were identified for the pilot projects, based on the prevalence of diabetes, on involvement in the National Programme for Prevention and Control of Cardiovascular Diseases, Cancers, Diabetes and Stroke, on the presence of mentoring institutions willing to play a role in building capacity, mentoring and management, and on geographical spread. As with the retinopathy of prematurity pilot project, WHO’s health system framework was used during development (Table 2).

## Pilot project implementation

During the first two years of the pilot projects, the focus was on establishing sustainable and scalable systems for controlling the two conditions. This was achieved. Both projects encountered challenges associated with an increased workload, the shortage of skilled staff, including trained ophthalmologists, and a lack of equipment. The pilot projects were rolled out sequentially, implementation started first in districts with better capacity. In many states, coming to an agreement with the state government took time. However, it was worthwhile, because it ensured local engagement and sustainability. Local elections and natural disasters delayed implementation in some states. The desired outcome was that the pilot projects should become integrated into the public health system and should over time be extended to other districts in participating states and to other non-participating states. This required substantial advocacy as it was not easy to convince some state governments to participate, because of competing health priorities. Moreover, some state and district coordination committees did not meet regularly. Partner organizations responsible for project implementation are being supported in their efforts to convene these meetings as active government engagement is critical to success.

For retinopathy of prematurity, specific challenges included: (i) poor coordination between neonatal care and eye-care services; (ii) a lack of synergy and trust between the public sector (where most preterm babies are treated) and the not-for-profit and private sectors, which have the expertise; and (iii) limited awareness of the risk of retinopathy of prematurity.^37^ In addition, states were initially reluctant to send district ophthalmologists for 8 to 10 weeks’ training in retinopathy of prematurity. Training, supportive supervision and encouragement helped overcome these problems. At the outset, mentoring partners found it difficult to persuade states to convene state coordination committee meetings.

For diabetic retinopathy, uptake of screening was poor initially due to a lack of awareness among people with diabetes of the seriousness of the disease and its complications and of the need for repeated visits to different specialists and for lifelong medication. Other challenges included: (i) poor communication between physicians treating people with diabetes and ophthalmologists; (ii) a reluctance to task-share; and (iii) the absence of structured follow-up mechanisms. The lack of affordable anti-vascular endothelial growth factors was also a challenge in the beginning, but later some state governments made budgetary allocations for supplies. In addition, implementation was hampered in many districts, because dedicated personnel and clinic space were not available. Another challenge was tracking people with diabetes through the care pathway, from noncommunicable disease clinics, through to eye-care providers. Project software was not used optimally in some states. In some districts, eye-care personnel, such as ophthalmic assistants, who could be trained to screen for diabetic retinopathy were not available.

## Sustainability

The retinopathy of prematurity pilot project has already led to major benefits: there is greater awareness of the need for systematic screening and screening has been introduced in non-participating districts. Moreover, some state governments have provided equipment to non-participating special newborn care units and have adopted the same hub-and-spoke model for expanding retinopathy of prematurity services. The pilot project also gave nongovernmental organizations the confidence to offer support to the public health sector; two non-participating states, encouraged by increased awareness and technical support, have started retinopathy of prematurity programmes with the help of nongovernmental organizations. Many other non-participating states have also requested technical support to start retinopathy of prematurity programmes. In addition, UNICEF, the agency providing technical support for child health at national and state levels, has included retinopathy of prematurity in their next five-year plan. The National Programme for Control of Blindness is also planning to provide funds for public sector medical schools to strengthen their capacity to screen and treat retinopathy of prematurity and to expand services. Information, education and communication materials developed for the project in local languages have been distributed widely to both health-care providers and parents. Software developed for the retinopathy of prematurity pilot project is being used for the follow-up, referral, coordination and monitoring of services. Moreover, the state coordination committees established for the project are providing platforms for participants to share details of their concerns and successes and to seek the support of policy-makers.

Awareness of the need for systematic screening for diabetic retinopathy has increased over time. Many state governments have introduced service delivery models similar to that used in the diabetic retinopathy pilot project in non-participating districts. In addition, the pilot project also attracted the attention of the for-profit private sector. For example, in Andhra Pradesh State, a leading for-profit hospital chain entered into an agreement with the state government to install fundus cameras in all primary care facilities and to provide comprehensive eye care, including screening for diabetic retinopathy. In Odisha State, the government has earmarked resources to develop the skills and infrastructure needed for comprehensive eye care, including screening for diabetic retinopathy. Similar comprehensive screening programmes are being planned in Gujarat, Karnataka, Kerala and Telangana States. The National Programme for Control of Blindness is also planning to involve states not covered by the pilot project. Information, education and communication materials are available in 10 local languages and the tablet computers provided in the projects to register people with diabetes are being used in six states, particularly for arranging follow-ups. The project’s district coordination committees provide fora where participants can share details of successes and concerns with policy-makers and programme managers in the public health system, which has increased the states’ sense of responsibility. Finally, implementation reviews are conducted regularly to help project partners learn from each other.

Our pilot projects for controlling diabetic retinopathy and retinopathy of prematurity have demonstrated that screening and treatment can be successfully integrated into the public health system using a partnership approach in a way that ensures sustainability and fosters comprehensive eye care. This approach could also be used for other potentially blinding conditions. Moreover, there are indications that the Government of India’s health plans are dynamic and respond to evidence provided by such projects. For example, the priorities of the National Programme for Control of Blindness have evolved and adapted to epidemiological trends over the past four decades. Initially, the emphasis was on trachoma and vitamin A deficiency. Then, based on evidence from national surveys, cataracts became a priority.^38^^–^^42^ Recent studies have found a decline in the prevalence of cataract blindness and national plans now support a comprehensive approach to eye care.^43^^–^^45^
